# Comparative Assessment of Sperm Morphology in Liquid-Preserved Boar Semen Using Cytological Stains

**DOI:** 10.3390/ani15182737

**Published:** 2025-09-19

**Authors:** Annika Braune, Axel Wehrend, Johannes Kauffold, Abbas Farshad

**Affiliations:** 1 Veterinary Clinic for Reproductive Medicine and Neonatology, Justus-Liebig-University of Giessen, 35392 Giessen, Germany; annika-braune@web.de (A.B.);; 2 Clinic for Ruminants and Swine, Faculty of Veterinary Medicine, University of Leipzig, 04103 Leipzig, Germany

**Keywords:** boar, fresh, storage, spermatozoa, staining method, light microscopy

## Abstract

Accurate assessment of sperm morphology is essential for artificial insemination using liquid-preserved boar semen. This study compared nine commonly used staining techniques, eosin, eosin–nigrosin, Diff-Quick^®^, Hemacolor^®^, Sangodiff-G^®^, Spermac^®^, Formol–Citrate–Rose Bengal stain, Testsimplets^®^, and Methyl Violet, based on morphological assessment, cost, time efficiency, and storage stability. Each staining method was applied to 36 slides, totaling 324 samples, and evaluated four times each (1296 evaluations). Slides were analyzed four times: immediately after staining and after 1 day, 1 week, and 3 months of storage. The results indicated that Eosin was the fastest and most cost-effective method, providing strong contrast, though it increased detection of structural alterations. Eosin–nigrosin offered detailed morphology but formed colored crystals over time. Diff-Quick^®^ and Hemacolor^®^ showed good initial performance, but Hemacolor^®^ lost pigment clarity after 3 months (*p* = 0.0273). Sangodiff-G^®^ had poor contrast and reduced detection of abnormalities (*p* = 0.00229). Spermac^®^ delivered high contrast but was time-consuming. Formol–Citrate–Rose Bengal stain required extensive preparation and showed significant post-storage changes (*p* < 0.0001). Testsimplets^®^, despite their high cost, suffered from declining interpretability (*p* < 0.0001). Methyl Violet lacked sufficient resolution and was highly unstable over time (*p* < 0.0001). In conclusion, Eosin emerged as the most practical and economical staining method for routine morphological evaluation of liquid-preserved boar semen. While eosin–nigrosin was also effective, its storage instability limits broader application. Other methods showed specific weaknesses, emphasizing the need to tailor stain selection to laboratory goals and constraints.

## Simple Summary

Staining techniques are crucial for evaluating boar sperm morphology. This study compared nine commonly used methods, including eosin, eosin–nigrosin, Diff-Quick^®^, and Spermac^®^, based on morphological assessment, cost, time efficiency, and storage stability. Each method was applied to 36 slides, totaling 324 slides, with each slide evaluated four times (1296 evaluations). Eosin proved to be the fastest and most cost-effective method, despite causing more structural alterations. Eosin–nigrosin provided good morphological details but showed crystal formation. Methyl Violet and Testsimplets^®^ had significantly lower interpretability (*p* < 0.0001). Spermac^®^ offered strong contrast but was time-consuming. Overall, eosin emerged as the most practical option for routine morphological evaluation of boar semen, while other methods showed notable limitations.

## 1. Introduction

The significance of sperm morphology in semen quality and male fertility is well-recognized [1,2]. Normal sperm morphology is essential for natural conception, in vitro fertilization (IVF), embryonic development, and clinical pregnancy outcomes [3,4,5]. Sperm morphology assessment has gained prominence in manuals on semen examination and processing [6,7]. Assessing sperm morphology helps predict fertilization potential, as detailed analyses offer essential insights into reproductive capacity [8,9,10]. In this context, stained spermatozoa examined under light microscopy remain fundamental to sperm quality assessment [11]. Various staining methods for porcine semen provide unique insights into morphology, vitality, and acrosome integrity, among other critical parameters. For example, eosin staining, first documented by Lasley et al. [12], evaluates sperm vitality based on plasma membrane permeability. Additionally, eosin–nigrosin staining, as used by Hancock [13], enhances the differentiation of stained and unstained sperm heads and assesses acrosomal integrity. Similarly, the Diff-Quick^®^ staining method, introduced by Kruger et al. [14], allows for quick and standardized analysis, offering reliable insights into sperm morphology and enabling efficient automated sperm cell analysis.

Hemacolor^®^ staining is a rapid and effective three-step procedure that employs methanol for fixation, eosin G to stain basic cellular structures, and a blue mixture of azur B and methylene blue to target acidic components. This technique produces distinct contrast, making it highly suitable for morphological evaluation of boar spermatozoa [15,16]. Complementing this approach, Sangodiff-G^®^, a method analogous to classic Giemsa staining, is applied to air-dried smears and offers a reliable means of assessing cellular morphology [17]. In contrast, Spermac^®^ utilizes a dichromatic staining strategy specifically designed to evaluate acrosomal integrity; it proves particularly valuable for post-thaw sperm quality assessment following cryopreservation [18,19]. Formol–Citrate–Rose Bengal stain combines formaldehyde, citrate, and Bengal pink for detailed sperm morphology analysis [20]. Testsimplets^®^, dye coated slides, differentiate sperm morphology and have shown variable results [21,22]. Methyl Violet has been used for assessing structural abnormalities in boar sperm and for detecting Cryptosporidium [23,24,25]. The Wells-Awa method is most effective for acrosome staining, while Farelly and Cerovsky techniques are efficient for sperm abnormalities [26]. Trypan Blue with Giemsa staining differentiates between live and dead sperms and accurately identifies acrosomes [27,28]. Fluorescent dyes like Propidium Iodide and Carboxyfluorescein Diacetate evaluate sperm functionality in fluorescence microscopy [29]. SpermBlue^®^ stain is universal, fast, easy to prepare, and suitable for automated semen analysis systems, showing differences in sperm components at various blue intensities [30].

Recent studies have highlighted the influence of staining protocols and storage conditions on sperm morphometry. Stain selection has been found to significantly alter quantitative morphology [31], which underscores the need for standardization when comparing results across laboratories [32]. Storage temperature also affects staining outcomes, with eosin–nigrosin producing the smallest head and tail dimensions at 17 °C, while SpermBlue^®^ and eosin–gentian yield larger measurements over time [33,34]. Furthermore, SpermBlue^®^ staining has been associated with a higher proportion of acrosome-reacted spermatozoa, peaking at 96 h, thereby offering a valuable complement to conventional acrosomal assessment methods [35,36].

Research in spermatology has demonstrated that various staining methods are essential for visualizing distinct structural features of sperm cells across different species. However, comparative evaluations of these techniques, particularly in relation to boar sperm morphology, remain limited. Therefore, the primary objective of this study was to identify the most effective staining methods for the light microscopic evaluation of sperm morphology in liquid-preserved porcine semen. The staining techniques evaluated, eosin, eosin–nigrosin, Diff-Quick^®^, Hemacolor^®^, Sangodiff-G^®^, Spermac^®^, Formol–Citrate–Rose Bengal stain, Testsimplets^®^, and Methyl Violet, were selected based on their previous application or reported effectiveness in morphological assessment. Beyond immediate morphological clarity, the study also assessed each method in terms of cost-efficiency, time requirements, and stability during prolonged slide storage. Long-term preservation of stained slides is particularly important for retrospective analyses, diagnostic validation, and quality control. In both clinical and research contexts, archived slides can serve as critical legal documentation, especially in cases involving breeding disputes or regulatory reviews. Thus, evaluating staining methods for their reliability over time for morphological evaluation is essential to ensure consistent diagnostic value and practical applicability.

## 2. Materials and Methods

### 2.1. Chemicals

This study utilized chemicals and reagents from Menzel Gläser (Braunschweig, Germany), Sigma-Aldrich (Darmstadt, Germany), Minitüb (Tiefenbach, Germany), Waldeck (Münster, Germany), BEG Schulze Bremer GmbH (Dülmen-Rorup, Germany) in Germany, and Medion Diagnostics (Düdingen, Switzerland).

### 2.2. Experimental Design and Procedures

This study was conducted at the Clinic of Reproductive Medicine and Neonatology, Justus Liebig University, Giessen, to assess the effectiveness of various staining techniques in detecting morphological changes in liquid-preserved porcine semen. Twelve semen samples were collected from the Artificial Insemination Center Union of Hesse eG in Darmstadt, Germany, using only commercially available materials to ensure practical applicability. Only semen with motility and normal morphology ≥ 95% were used for the study. All samples were extended with BTS Gentamycin and originated from Pietrain and German Landrace boars aged approximately 12 to 25 months. Each semen sample was divided into three aliquots and incubated overnight under different conditions to simulate typical handling scenarios: refrigeration at 6 °C (cold storage), incubation at 38 °C in a water bath (WNB 45, Memmert, Schwabach, Germany), and storage at 18 °C in a dimly lit environment (room temperature). After 24 h, smears were prepared from each aliquot and stained using one of nine protocols: eosin, eosin–nigrosin, Diff-Quick^®^, Hemacolor^®^, Sangodiff-G^®^, Spermac^®^, Formol–Citrate–Rose Bengal stain, Testsimplets^®^, or Methyl Violet. To compare the performance of these nine staining methods for assessing porcine sperm morphology, ejaculates from 12 boars were used. Importantly, the evaluation was performed on the prepared and stained smears rather than on fresh semen. The slides were examined at four defined time points to assess their archival stability: immediately after staining (Time 1), after one day (Time 2), after one week (Time 3), and after three months (Time 4). To ensure robust representation and statistical reliability, each of the nine staining methods was applied to 36 distinct slides. This resulted in a total of 324 unique slides across all methods (9 methods × 36 slides). To evaluate consistency and reproducibility, each slide was independently assessed four times, yielding a comprehensive dataset of 1296 evaluations (9 methods × 36 slides × 4 evaluations). Furthermore, the term analyzable denotes the proportion of slides that demonstrate sufficient staining quality and visual clarity to permit reliable evaluation of sperm morphology. This metric, therefore, reflects the overall usability of the sample for analytical purposes. Morphological evaluation was performed on 200 spermatozoa per slide using the “Assistant” Counter AC-15 (Karl Hecht, Sondheim, Germany), based on the criteria established by Hancock [13].

To assess morphological changes in boar sperm cells following liquid preservation, defined evaluation criteria were applied to distinct sperm regions. The head and acrosome were examined for shape abnormalities (e.g., round, lanceolate, pear-shaped), size deviations (too large or too small), double heads, detachment, absence of the acrosome, and the presence of vacuoles. The midpiece and principal piece were assessed for asymmetry, fractures, thickening or narrowing, loops, coiling, sharp bends, and breakage. Secondary and tertiary defects, including detached heads and structural anomalies of the midpiece, principal piece, and tail, were also recorded. Additionally, cytoplasmic droplets were noted when present. To complement the morphological analysis, staining quality was assessed based on three parameters: color intensity, detail recognition, and contrast, modified from the method described by [32]. Each parameter was rated on a 5-point scale (1 = lowest, 5 = highest), with qualitative descriptors in parentheses provided for reference. Reported scores represent the means of two independent, blind evaluations per slide. This comprehensive approach ensured reliable assessment of both sperm integrity and staining efficiency. Slides were stored in a Rotilabo^®^ slide box (Carl Roth, Karlsruhe, Germany) at room temperature (approximately 22 °C) in a dark cabinet and re-evaluated at the aforementioned time. These follow-ups focused on changes in slide interpretability to determine the archival suitability of the different staining methods.

Following the morphological evaluation, the time required for both preparation and microscopic assessment of stained slides was systematically recorded for each staining method. Preparation included all steps necessary to produce a smear ready for microscopy, while evaluation comprised the time spent assessing sperm morphology and, where applicable, viability. Total processing time per slide was calculated as the sum of preparation and evaluation times. Based on these measurements, labor costs were calculated using an hourly wage of €25 for a certified veterinary technician, and material costs included reagents, slides, coverslips, and consumables. Total costs per slide were obtained as the sum of material and labor costs. A sensitivity analysis was performed to account for ±10% variations in material costs and the inclusion or exclusion of labor costs. Reference micrographs for each staining method and the associated cost and time data were collected under standardized conditions, providing a practical estimate of the overall resource requirements for routine morphological assessment of liquid-preserved boar spermatozoa.

### 2.3. Staining Techniques

Nine different staining methods were employed to evaluate specific morphological features of liquid-preserved boar spermatozoa. Each staining protocol was applied according to manufacturer instructions or established literature references [32]. Target structures, preparation procedures, and diagnostic purposes vary among stains due to their distinct staining principles. To complement the morphological analysis, staining quality was quantitatively assessed using three parameters: color intensity, detail recognition, and contrast. Each parameter was rated on a 5-point scale (1 = lowest, 5 = highest), with qualitative descriptors provided in parentheses. Reported scores represent the means of two independent, blind evaluations per slide. These scores serve as the standardized unit for comparing staining effectiveness across all methods.

#### 2.3.1. Eosin Staining

A 2% eosin solution was prepared by dissolving 2 g of Eosin B and 3 g of trisodium citrate dihydrate in 100 mL of distilled water. For each smear, 10 µL of semen was mixed with 10 µL of eosin solution on a pre-warmed microscope slide, spread using a second slide at a 45° angle, and air-dried. Slides were examined at 400× magnification. Dead spermatozoa absorbed the dye (pink to red), while live sperm remained unstained, enabling viability assessment, which was recorded as the observed percentage of stained cells [32,34].

#### 2.3.2. Eosin–Nigrosin Staining

Unlike other stains, eosin–nigrosin simultaneously assesses viability and morphology. The staining solution was prepared by dissolving 0.67 g of Eosin Y and 0.9 g of sodium chloride in 100 mL of distilled water, followed by the addition of 10 g of nigrosin. The solution was boiled, cooled, filtered, and stored in a dark bottle. Equal volumes (10 µL) of semen and stain were mixed on a slide and evaluated immediately at 1000× magnification with immersion oil. Live spermatozoa appeared light against a dark background, while dead cells were stained pink to red by eosin. Viability was recorded as the proportion of unstained cells. Morphology and staining quality were scored using the 5-point scale.

#### 2.3.3. Diff-Quick^®^ Staining

The Diff-Quick^®^ kit (Medion Diagnostics, Düdingen, Switzerland) was used for morphological assessment. The procedure included three steps: fixation in methanol-based Diff-Quick Fix for 15 s; staining with Diff-Quick I (eosin G) for 10 s; and counterstaining with Diff-Quick II (azure A, methylene blue) for 5 s. Slides were briefly rinsed, air-dried, and evaluated at 400× magnification. The head stained blue, cytoplasm pink to red, and acrosomal detail was clearly distinguishable. Staining quality was recorded using the 5-point scale.

#### 2.3.4. Hemacolor^®^ Staining

Using the Hemacolor^®^ kit (Merck Chemicals, Darmstadt, Germany), a standardized three-step staining process was followed. Slides were first fixed by dipping five times (1 s each) in Solution 1 (methanol). This was followed by red staining with three dips in Solution 2 (eosin G), and then blue staining with six dips in Solution 3 (azure B and methylene blue). After each step, excess stain was gently removed by vertically blotting the slide on absorbent paper. The reverse side was rinsed with water and air-dried before evaluation. Evaluation at 400× magnification allowed scoring of color intensity, detail recognition, and contrast using the 5-point scale. Sperm heads appeared blue with clearly defined nuclear and acrosomal regions, while the cytoplasm-stained light pink, enabled detailed morphological assessment.

#### 2.3.5. Sangodiff-G^®^ Staining

Sangodiff-G^®^ film-coated slides (Merck Chemicals) containing azure, eosin, and methylene blue were used. After methanol fixation and air-drying, a buffer was applied and the film activated for 10 min. Slides were rinsed, dried, and examined at 400× magnification. The acrosome-stained deep red, while cytoplasmic components appeared light pink, and staining quality was scored using the 5-point scale.

#### 2.3.6. Spermac^®^ Staining

Spermac^®^ provided detailed differentiation of sperm structures through a multi-step protocol. After fixation in 4% formalin for 5–6 min, smears were stained sequentially: (1) red stain (Rose Bengal, neutral red, ethanol), (2) light green stain (Pyro-nin Y, molybdophosphoric acid, ethanol), and (3) dark green stain (Janus green, Fast Green FCF), each for 1 min. Slides were gently rinsed and blotted between steps. At 1000× magnification, acrosome (dark green), nucleus (red), equatorial segment (light green), and midpiece/tail (green) were scored for staining quality using the 5-point scale.

#### 2.3.7. Formol–Citrate–Rose Bengal Staining

Rose Bengal is a penetrative dye that stains all sperm membranes after fixation, regardless of viability. The resulting pink coloration is a staining artifact and not a reliable marker of non-viable cells. In this study, it was used solely to enhance morphological visualization, not to assess sperm vitality. For staining, a solution was prepared by mixing 20 mL of semen with 0.3 mL of Formol–Citrate–Rose Bengal stain reagent, composed of 2.9 g trisodium citrate dihydrate, 4 mL of 35% formaldehyde, and 0.156 g Rose Bengal dissolved in 100 mL of distilled water. A drop of the mixture was placed on a glass slide, covered with coverslip, and examined after 30 min under oil immersion at 1000× magnification. Under these conditions, non-viable spermatozoa typically appeared pink to red, while viable cells remained unstained. Due to the non-selective nature of the dye, this distinction is not fully reliable; therefore, staining quality was additionally scored using a 5-point scale for color intensity.

#### 2.3.8. Testsimplets^®^ Staining

Testsimplets^®^ (Waldeck, Münster, Germany) are commercially prepared slides coated with dry dyes such as new methylene blue and cresyl violet acetate. A 10 µL drop of semen was placed on a coverslip, which was then pressed onto the stained slide and left in contact for 5–15 min. After incubation, slides were evaluated at 1000× magnification using immersion oil, scoring of morphology, and staining quality using the 5-point scale. Spermatozoa exhibited well-defined staining, with nuclei appearing blue to violet and acrosomal regions lightly stained, allowing for clear morphological assessment.

#### 2.3.9. Methyl Violet Staining

This stain (BEG Schulze Bremer GmbH, Dülmen-Rorup, Germany) included methyl violet and a sperm-inactivating agent. Equal volumes (10 µL) of semen and stain were mixed on a slide, covered with a coverslip, and evaluated after 10 min at 400× magnification. Head staining intensity correlated with viability, and staining quality was scored on the 5-point scale.

### 2.4. Statistical Methods

Statistical analysis was conducted using Microsoft^®^ Excel 2007 for data organization and R software (Version 4.3.1; R Core Team, 2023) for advanced modeling, specifically applying the *glmmPQL* function. A Poisson regression model within a Generalized Linear Mixed Model (GLMM) framework was used to evaluate how different staining methods and storage durations influenced the frequency of sperm with pathomorphological abnormalities. The model accounted for repeated measures by including random effects. The significance of effects was assessed using Wald chi-square tests, with *p*-values ≤ 0.05 considered statistically significant and *p*-values ≤ 0.01 considered highly significant.

## 3. Results

### 3.1. Smear Quality of Staining Methods and Overall Morphology Results

The results presented in Table 1 show that eosin, eosin–nigrosin, Diff-Quick^®^, Spermac^®^, and Hemacolor^®^ provided consistently strong contrast at Time 1 (immediate). Among these, eosin, eosin–nigrosin, and Spermac^®^ offered balanced or saturated color intensity along with clear detail recognition. Diff-Quick^®^ and Hemacolor^®^ displayed acceptable levels of detail, although Hemacolor^®^ was noted for its faint color intensity.

As presented in Table 2, the proportion of morphologically abnormal sperm cells was slightly higher (26.44%) compared to the average (25.59%), with a high number of cytoplasmic droplets (10.33%, average 5.39%). Ultimately, the detailed findings and assessment quality across different times for the limited assessable stains such as Sangodiff-G^®^, Formol–Citrate–Rose Bengal stain, Testsimplets^®^, and Methyl Violet are presented in Table 3. Hemacolor staining maintained good contrast, although it exhibited some issues by the fourth time, while Sangodiff-G^®^ staining showed a significant decrease in evaluation quality over time, with notable issues in color intensity and background consistency, Formol–Citrate–Rose Bengal stain consistently performed well across all times. Meanwhile, Testsimplets^®^ and Methyl Violet staining methods demonstrated varying degrees of performance, with notable declines in examination quality at the later times.

**Figure 1:**
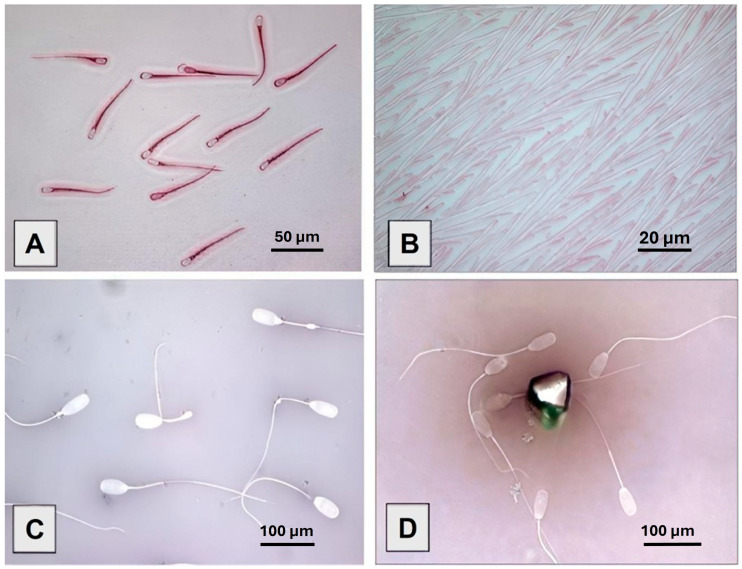
Presents a series of microscopic images illustrating various biological samples, including liquid-preserved boar semen stained with eosin and eosin–nigrosin. Panels (**A**,**B**) show samples stained with eosin and observed at 400× magnification using a 40× objective. Panel (**A**) displays elongated, pink-stained structures resembling Demodex-like elements within hair follicles, while Panel (**B**) reveals spindle-shaped fibroblasts aligned in parallel, typical of cultured connective tissue. Panels (**C**,**D**) depict sperm cells stained with eosin–nigrosin and examined at 1000× magnification using a 100× oil immersion objective. Panel (**C**) shows sperm cells with oval heads and long flagella dispersed across the field, and Panel (**D**) captures a cluster of sperm surrounding a central, cone-shaped microinjection device. All micrographs include visible scale bars ((**A**): 50 µm, (**B**): 20 µm, (**C**): 100 µm, (**D**): 100 µm), and exposure settings were kept consistent across panels to ensure comparability.

Regarding morphology, the results showed that all eosin-stained samples demonstrated fair color intensity, clear detail visibility, good contrast, and a distinct background during the first three times (Figure 1A). At Time Point 2, 30% of the samples and at Time Point 4, 41.67% displayed crystal deposits on sperm (Figure 1B), which complicated the pathomorphological assessment. By the three-month mark (Time Point 4), the stained samples revealed a slightly elevated incidence of morphological abnormalities (26.53%) in comparison to the average (25.59%), with a notable proportion of sperm exhibiting detached acrosomes (4.53%) and fractured midpieces (1.69%). Furthermore, isolated sperm heads were observed with the highest frequency (3.83%). Regarding the results of eosin–nigrosin staining, all 36 samples exhibited fair color intensity, clear details, contrast, and a clear background at all time points (Figure 1C). At Time Points 2–4, 16.67% developed large color crystals (Figure 1D). Additionally, at Time Point 4, 50% of the samples showed parallel white stripes in the homogeneous gray background. As presented in Table 2, the proportion of morphologically abnormal sperm cells was slightly higher (26.44%) compared to the average (25.59%), with a high number of cytoplasmic droplets (10.33%, average 5.39%). Ultimately, the detailed findings and assessment quality across different time points for the limited assessable stains such as Sangodiff-G^®^, Formol–Citrate–Rose Bengal stain, Testsimplets^®^, and Methyl Violet are presented in Table 3. Hemacolor staining maintained good contrast, although it exhibited some issues by the fourth time point. While Sangodiff-G^®^ staining showed a significant decrease in evaluation quality over time, with notable issues in color intensity and background consistency, Formol–Citrate–Rose Bengal stain consistently performed well across all time points. Meanwhile, Testsimplets^®^ and Methyl Violet staining methods demonstrated varying degrees of performance, with notable declines in examination quality at the later point.

**Figure 2:**
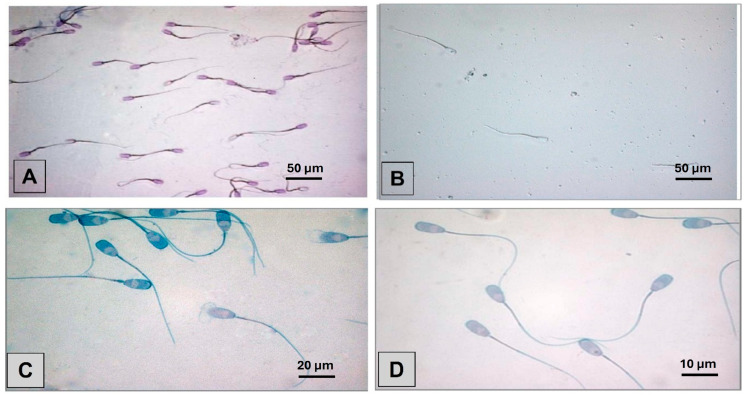
Illustrates the microscopic visualization of liquid-preserved boar semen stained with Diff-Quick^®^ and Spermac^®^, emphasizing temporal changes in staining characteristics. Panels (**A**,**B**) show samples treated with Diff-Quick^®^ and observed at 400× magnification using a 40× oil immersion objective. Panel (**A**) captures the initial appearance of color particles at Time 1, while Panel (**B**) reflects their distribution after three months (Time 4). In contrast, Panels (**C**,**D**) present samples stained with Spermac^®^ and examined at 1000× magnification using a 100× oil immersion objective. Panel (**C**) reveals the original color intensity at Time 1, whereas Panel (**D**) demonstrates a marked fading of stain intensity at Time 4. All micrographs include visible scale bars—50 µm in Panels (**A**,**B**), 20 µm in Panel (**C**), and 10 µm in Panel (**D**)—and exposure settings were standardized across all panels to ensure consistency in image interpretation.

Regarding the Diff-Quick^®^ staining, the results indicated a pale color intensity, measured detail, and satisfactory contrast (Figure 2A). In Table 3, at Time 4, 33.33% of the samples exhibited fading color, and 16.67% showed particle deposits on the sperm tails. Detection of cytoplasmic droplets was recorded at 5.28%, which is consistent with the overall average of 5.39% across all staining methods. Additionally, 16.67% of the samples displayed indistinct sperm heads and acrosomes, while 8.33% were classified as unclear at Time 4. Furthermore, the results of Spermac^®^ as a quick and reliable method for evaluating porcine sperm cells were fully evaluable at Time 1 and exhibited good color intensity (Figure 2C), sharpness, and contrast showing a 1.14% rate compared to the 0.33% average of other methods. However, the color intensity significantly faded at Time 4 (Figure 2D). The staining method, Methyl Violet, specifically marketed for boar semen, lacks scientific studies for porcine spermatozoa. The following product instructions did not yield satisfactory results. The staining showed pale intensity, poor detail visibility, and low contrast. Overlapping sperm cells with lance-shaped heads dominated the image, and droplet formation led to looped sperm cells after one day. After a week, the cells settled in multiple layers, and evaluation at later times was impossible due to dye droplet contraction.

In a qualitative assessment of the results according to the minimum requirements for porcine semen portions (a maximum of 25% morphologically altered spermatozoa, including cytoplasmic droplets, and a tolerable counting deviation of 5%), the staining-based evaluations were found to be slightly above the acceptable threshold. The elevated proportion of morphologically altered spermatozoa is most likely due to the deliberate ordering of lower-quality semen from the insemination station Darmstadt/Griesheim of the Zucht- und Besamungsunion Hessen eG, in order to better demonstrate the ability of the different staining methods to analyze pathomorphological changes.

### 3.2. Pathomorphological Differences Across Staining Methods and Storage Times

**Figure 3:**
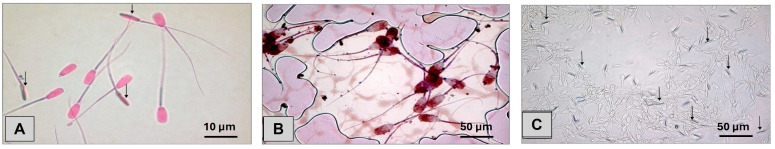
Demonstration of liquid-preserved boar semen stained using various methods and viewed under magnification with immersion oil. Panel (**A**) shows samples stained with Formol–Citrate–Rose Bengal at 1000× magnification using a 100× oil immersion objective, revealing lance-shaped structures at Time 1 and droplet formations at Time 4. The corresponding scale bar is 10 µm, appropriate for the high-resolution visualization of fine morphological changes. Panel (**B**) presents samples stained with Testsimplets^®^ at 400× magnification using a 40× oil immersion objective, highlighting droplet formation and a yellowish, blurred appearance at Time 4. The scale bar is 50 µm, suitable for mid-range magnification and broader field observation. Panel (**C**) displays samples stained with Methyl Violet at 400× magnification using a 40× oil immersion objective, showing lance-like structures at Time 1 and droplet formation at Time 4. The scale bar is 50 µm, consistent with the magnification and structural detail. All micrographs include visible scale bars and were captured under standardized exposure settings to ensure consistency in image interpretation across panels. Arrows indicate the morphological abnormalities (e.g., head or tail defects) observed in spermatozoa.

A comparative analysis of pathomorphological findings across different storage temperatures revealed significant differences (*p* < 0.0001) when evaluating all staining methods except Methyl Violet (Table 2). Methyl Violet was excluded from statistical analysis due to consistently poor staining quality, characterized by pale coloration, low detail visibility, and poor contrast in all 36 samples, which led to unassessable or distorted sperm morphology, especially at Time 1, where lance-shaped formations persisted for up to four hours post-preparation (Figure 3C). At Time 2, although color intensity was comparable to Diff-Quick^®^ and Hemacolor^®^, substantial droplet formation led to overlapping and distorted sperm cells. By Time 3, droplet contraction and cell layering further hindered evaluation. Among the other staining methods, morphological differences were evident. Testsimplets^®^ showed the highest proportion of abnormal sperm at 31.81%, while Spermac^®^ yielded the lowest at 18.31%, with an overall average of 25.59%. Irregular head shapes were rare across all methods (mean 0.52%, *p* = 0.721). The highest proportion of lance-shaped heads was observed with Formol–Citrate–Rose Bengal stain (7.97%, *p* < 0.0001). Pear-shaped heads were only noted with Testsimplets^®^ (0.22%) and absent in both eosin and eosin–nigrosin stains *(p* = 0.004). Duplicate heads were most frequently seen with Hemacolor (0.14%, *p* = 0.0353). Acrosomal integrity also varied significantly: Sangodiff-G^®^ and Testsimplets^®^ had the highest rates of missing acrosomes (1.19% and 1.14%, respectively; *p* < 0.0001), while eosin (4.53%) and Sangodiff-G^®^ (6.31%) showed increased rates of acrosome detachment (*p* < 0.0001). Rolled-up principal pieces ranged from 6.92% with Diff-Quick^®^ to 10.22% with Testsimplets^®^ (*p* = 0.0014). Additionally, cytoplasmic droplets at the neck were more frequent in samples stained with eosin–nigrosin (6.72%) and Testsimplets^®^ (7.5%, *p* < 0.0001).

Furthermore, Table 4 shows the statistical evaluation of sperm pathomorphological abnormalities at Time Point 1 across three storage temperatures (6 °C, 18 °C, and 38 °C). Overall, no significant differences in the total proportion of abnormal spermatozoa were found between temperatures (*p* = 0.908). However, specific abnormalities varied: irregular head shapes were significantly more frequent at 6 °C (0.56%) than at 38 °C (0.33%; *p* = 0.047). Pear-shaped heads were also more common at 6 °C (0.08%; *p* < 0.0001). Double heads peaked at 18 °C (0.05%; *p* = 0.0238). Rolled principal pieces were more prevalent at 6 °C and 38 °C (0.83%) than at 18 °C (0.48%; *p* = 0.0191). Plasma droplets on the principal piece were highest at 18 °C (0.05%; *p* = 0.0007). Acrosome detachment was slightly elevated at 38 °C (2.21%), though not significant (*p* = 0.18). To identify the most suitable staining methods, smear quality and analyzability were assessed over four times. At Time 1, all methods except Methyl Violet provided good clarity; Methyl Violet was excluded due to poor performance. By Time 2, most methods, particularly Formol–Citrate–Rose Bengal stain, Spermac^®^, and eosin–nigrosin, retained staining quality. In contrast, Testsimplets^®^ exhibited increasing background interference, which worsened over time. By Time Point 4, its analyzability had declined sharply to just 11.11%. Hemacolor and Sangodiff-G^®^ also showed progressive loss of clarity. Only Formol–Citrate–Rose Bengal stain and Spermac^®^ consistently maintained stable staining quality and clear sperm morphology throughout the observation period.

### 3.3. Time Consumption and Cost Analysis of Staining

Table 5 summarizes the differences in preparation, evaluation, and processing times for stained samples. Eosin and eosin–nigrosin were the fastest, taking around 8–10 min per slide, while Formol–Citrate–Rose Bengal stain, Methyl Violet, and Testsimplets^®^ were the slowest, ranging from 51 to 68 min. The vitality assessment showed that, on average, sperm viability was highest under refrigeration (RF: 88 ± 4%), intermediate at room temperature (RT: 84 ± 5%), and lowest in the water bath (WB: 72 ± 6%), and these values were incorporated into totals including vitality to allow direct comparison with staining methods that do not include a vitality step.

As presented in Table 6, costs varied, with Formol–Citrate–Rose Bengal stain being the most cost-effective at €0.15 per slide, while Testsimplets^®^ was the most expensive at €1.70 per slide. In addition, material costs per slide ranged from €0.15 for Formol–Citrate–Rose Bengal stain to €1.70 for Testsimplets^®^ (Table 6). Incorporating labor costs, which were calculated from the total processing time and an hourly wage of €25 for a certified veterinary technician, resulted in total costs ranging from €5.32 (eosin) to €29.89 (Testsimplets^®^). A sensitivity analysis considering ±10% variation in material costs and the inclusion/exclusion of labor costs is also presented in Table 6. Overall, the fastest stains (eosin and eosin–nigrosin) combined low material and moderate labor costs, while the slowest stains (Formol–Citrate–Rose Bengal, Methyl Violet, Testsimplets^®^) incurred the highest total costs. This comprehensive assessment of time and cost allows laboratories to consider both efficiency and expense when selecting appropriate staining methods.

## 4. Discussion

Evaluating liquid-preserved boar semen, commonly used in artificial insemination (AI), requires a detailed and standardized assessment of sperm quality to ensure that morphological abnormalities remain below the critical 25% threshold, beyond which fertility outcomes may be negatively affected [32]. In global AI practice, boar semen is typically used either immediately post-collection or after short-term storage (1–5 days) at 15–18 °C. However, storing semen below 15 °C has been shown to significantly increase the proportion of non-viable sperm, highlighting the pronounced thermal sensitivity of porcine spermatozoa [37]. In light of these challenges, this study systematically compared the performance of nine staining methods, eosin, eosin–nigrosin, Diff-Quick^®^, Hemacolor^®^, Sangodiff-G^®^, Spermac^®^, Formol–Citrate–Rose Bengal stain, Testsimplets^®^, and Methyl Violet, based on their effectiveness in evaluating sperm morphology, as well as their stability over time and under different storage temperatures (6 °C, 18 °C, and 38 °C), following the protocols outlined by Johnson et al. [38]. Additionally, time and cost efficiency were assessed to guide practical implementation in laboratory settings.

The major objective of this study was to examine how the passage of time influences the evaluability of stained slides and the preservation of sperm morphological detail. This is particularly important given that stained slides are often stored for later reassessment in laboratory workflows. Among the stains tested, eosin initially produced good contrast and effectively marked non-viable spermatozoa [32,39,40]. However, it demonstrated reduced staining intensity and contrast over time, leading to a higher incidence of observable abnormalities, such as isolated head defects and fractured midpieces. This suggests that while eosin is useful for immediate evaluation, its utility for delayed analysis is limited. By contrast, stains such as Formol–Citrate–Rose Bengal stain and Testsimplets^®^ preserved sperm morphology more consistently over time. Eosin–nigrosin, a well-established stain in porcine sperm evaluation [13,41,42,43,44,45], provided enhanced contrast and resolution, allowing for better visualization of subtle abnormalities compared to eosin alone. It revealed a lower proportion of abnormalities in the main piece (9.81%) compared to other stains (11.5%), although it tended to highlight cytoplasmic droplets (10.33%), which may reflect increased sensitivity to signs of sperm immaturity. Its capacity to visualize the acrosome, however, was inferior to that of Giemsa staining [44]. Interestingly, a combined application of eosin–nigrosin with Giemsa resulted in a differentiated staining pattern, where live sperm showed a pink post-acrosomal region and dead sperm appeared dark purple. This combination has been associated with increased detection of tail defects in other species [46,47,48,49], although such artifacts were not observed in porcine samples. Despite these initial advantages, eosin, eosin–nigrosin, Diff-Quick^®^, and Spermac^®^ all exhibited signs of deterioration over time, consistent with findings from previous studies [50,51]. Hemacolor^®^ also showed a gradual loss of stain intensity, which impaired the detection of fine morphological defects like cytoplasmic droplets. Sangodiff-G^®^ displayed similar but less pronounced degradation. In contrast, Formol–Citrate–Rose Bengal stain showed a reduction in observable head and acrosome defects over time, most likely due to stain fading rather than true morphological improvement [52,53]. Testsimplets^®^, in particular, suffered from a rapid decline in slide evaluability after only one week due to dye fading and droplet contraction. These changes compromised diagnostic accuracy and statistical reliability. These findings reaffirm the impact of storage duration on staining reliability, especially for dyes that diffuse or fade over time [36,49].

Stain choice has been shown to significantly reshape quantitative sperm morphology, reinforcing the importance of controlling staining protocols in comparative studies across laboratories [31]. Additionally, storage temperature has a notable impact on staining outcomes: at 17 °C, eosin–nigrosin produces the smallest head and tail dimensions, whereas eosin–gentian and SpermBlue yield the largest, with these morphometric changes developing over time [33]. This distinction clarifies the relationship between staining technique, the stability of treated preparations, and the drift in sperm morphology during liquid storage. Furthermore, a comparison of SpermBlue and eosin–gentian in the context of acrosome-reacted sperm revealed a higher proportion of reacted spermatozoa with SpermBlue, peaking at approximately 96 h, providing a useful counterpoint to the section on acrosomal assessment, particularly since SpermBlue was not among the evaluated staining methods [35]. Regarding the influence of storage temperature on sperm morphology, the observation shows a substantial effect on sperm morphology and stain performance. Deviations from the recommended range of 15–18 °C led to a marked increase in morphological abnormalities. Specifically, at 6 °C and 38 °C, rare abnormalities, such as irregularly shaped heads and pronounced cytoplasmic droplets, became more prominent, especially in samples stained with eosin, eosin–nigrosin, Diff-Quick^®^, Hemacolor^®^, and Methyl Violet. These findings align with prior research showing structural deterioration in porcine sperm outside optimal temperature conditions [42]. Interestingly, Sangodiff-G^®^ and Spermac^®^ demonstrated distinctive morphological responses under thermal stress, suggesting temperature-sensitive interactions between stain chemistry and sperm cell membranes. Similarly, Formol–Citrate–Rose Bengal stain and Testsimplets^®^ exhibited notable temperature-dependent changes, particularly affecting head and acrosome morphology. However, no uniform pattern could be established linking specific stains to temperature-induced defects, likely due to the complex interplay of biological and chemical variables. Vitality assessments reinforced the detrimental effects of elevated temperatures. While cooler storage conditions helped maintain sperm viability, exposure to 38 °C led to a substantial decline, again confirming the high thermosensitivity of porcine spermatozoa [42]. In some cases, unusual sperm shapes, such as lance- or needle-like forms—were observed. These were identified as artifacts from drying during slide preparation rather than true morphological defects [36]. Oberlender et al. [54] reported similar issues, noting increased tail defects in smear techniques compared to wet preparations, likely due to mechanical damage. Likewise, Czubaszek et al. [55] demonstrated that staining methods significantly affect sperm morphometry, with Sperm Blue better preserving the natural dimensions of the sperm head than eosin-based stains.

In regard to the time and cost efficiency of staining methods, the time and cost efficiency of each staining method varied notably. Eosin emerged as the most efficient, offering the shortest preparation time and lowest material costs, making it ideal for high-throughput settings. In contrast, Testsimplets^®^ was both the most time-consuming and the most expensive, primarily due to prolonged preparation and settling times. Methyl Violet and Formol–Citrate–Rose Bengal stain also required longer preparation periods, although the latter was the most cost-effective on a per-slide basis. Hemacolor^®^ showed variable pricing across suppliers, making direct cost comparisons difficult. Additionally, analysis duration was affected by microscope magnification; higher magnification levels slightly increased the time required per slide. These observations are in line with previous recommendations that emphasize optimizing laboratory workflows by balancing diagnostic precision, operational feasibility, and economic constraints [56]. Selecting an appropriate staining method should therefore consider not only diagnostic clarity and stability but also the logistical realities of laboratory capacity and resource availability.

## 5. Conclusions

This study systematically compared nine staining methods for porcine sperm analysis, considering morphological clarity, acrosomal assessment, slide stability, and cost-efficiency. Among the stains evaluated, eosin and eosin–nigrosin provided the most accurate morphological assessment and allowed rapid evaluation, making them the preferred choice for routine sperm quality analysis. However, their archival stability decreased over time. Spermac^®^ excelled in acrosomal demarcation and slide stability, making it particularly suitable for studies requiring long-term preservation and detailed acrosomal evaluation. Other stains, including Diff-Quick^®^ and Hemacolor^®^, showed weaker staining quality, while Sangodiff-G^®^ maintained morphology but was affected by residual dye. Testsimplets^®^ and Methyl Violet were limited by excessive dye deposition and artifacts. Storage temperature significantly influences sperm integrity, with higher temperatures causing more damage and lower temperatures being less harmful. Slide stability and staining quality also varied across methods. Eosin was the fastest method, while Testsimplets^®^ required the longest processing time. Some homemade stains remained effective and economical for routine use when considering both materials and labor. Despite these findings, the study has limitations: heterogeneous magnification across stains, variability in staining quality over time, and the exclusive use of high-quality ejaculates may reduce generalizability. However, future studies should include suboptimal semen samples, control for stain-temperature interactions, assess inter- and intra-observer agreement, and provide a more comprehensive economic evaluation to further enhance practical applicability. In summary, for accurate and rapid morphological evaluation, eosin and eosin–nigrosin are recommended, while Spermac^®^ is optimal when acrosomal detail and slide preservation are prioritized.

## Figures and Tables

**Table 1:** Description of various staining Methods at Time 1 (Immediate).

Staining Method	Color Intensity (1–5)	Detail Recognition (1–5)	Contras (1–5)
Eosin	3 (balanced color)	5 (clear detail visibility)	5 (marked difference)
Eosin–Nigrosin	3 (balanced color)	5 (clear detail visibility)	5 (marked difference)
Diff-Quick^®^	3 (balanced color)	4 (acceptable detail level)	5 (marked difference)
Spermac^®^	5 (saturated color)	5 (clear detail visibility)	5 (marked difference)
Hemacolor^®^	2 (faint color)	4 (acceptable detail level)	5 (marked difference)
Sangodiff-G^®^	2 (faint color)	4 (acceptable detail level)	2 (minimal tonal difference)
Formol–Citrate–Rose Bengal stain	5 (highly saturated)	5 (clear detail visibility)	5 (marked difference)
Testsimplets^®^	4 (saturated color)	5 (clear detail visibility)	4 (noticeable but not sharp)
Methyl Violet	2 (faint color)	3 (unclear details)	2 (minimal tonal difference)

Note: Data were organized and scored by two blind observers (400× objective). Means are reported. Statistical analysis used a Poisson GLMM (glmmPQL in R) with random effects; significance was tested by Wald chi-square (*p* ≤ 0.05 significant; *p* ≤ 0.01 highly significant). Color intensity, detail recognition, and contrast were rated on a 5-point scale (1 = lowest, 5 = highest). Qualitative descriptors in parentheses are provided for reference. The scores reflect the average of two separate, blinded evaluations conducted for each slide.

**Table 2:** Description of the comparison of pathomorphological results across storage temperatures at Time Point 1 (immediate analysis), showing the mean values (%) for each staining method (excluding methyl violet), the overall average (Ø), and the summed scores of relevant morphological subcategories per region (“Total”) for illustrative purposes. Statistical significance (*p*-values) is also indicated where applicable.

Criteria	E	EN	DQ	H	SG	S	FB	TS	Ø	*p*-Value
Abnormal sperm	26.53 ^a^	26.44 ^a^	22.56 ^b^	22.00 ^b^	18.31 ^c^	30.5 ^d^	26.58 ^d^	31.81 ^a^	25.59	<0.0001
Irregular head shape	0.36	0.58	0.47	0.47	0.53	0.72	0.47	0.53	0.52	0.721
Round head	0.53	0.33	0.36	0.36	0.39	0.5	0.44	0.39	0.41	0.926
Lanceolate head	0.06	0.03	0.03	0	0	0	7.97	3.86	1.49	<0.0001
Pear-shaped head	0	0.06	0	0.06	0	0	0.03	0.22	0.05	0.004
Double head	0	0.03	0	0.14	0.03	0.03	0.03	0	0.03	0.0353
Acrosome size variations	0	0	0	0	0	0	0	0.03	0.00	0
Absence of acrosome	0.11	0	0	0	1.19	1.14	0	0.17	0.33	<0.0001
Acrosome detachment	4.53	2.22	1.22	1.25	0.53	6.31	1.5	0.44	2.25	<0.0001
Acrosome with vacuoles	0	0.06	0.03	0	0	0.19	0.08	0	0.05	0.0007
Total head/acrosome	5.59 ^a^	3.31 ^b^	2.11 ^c^	2.28 ^c^	2.67 ^d^	8.89 ^d^	10.53 ^e^	5.64 ^a^	5.12	<0.0001
Asymmetrical midpiece	0	0	0	0	0	0	0	0	0	*
Broken midpiece	1.69	0.5	1.53	0.78	1.06	1.42	0.28	0.31	0.95	0.0009
Thickened/narrow midpiece	0.08	0	0.14	0	0	0	0.14	0.03	0.05	0.468
Total midpiece evaluation	1.78	0.5	1.67	0.78	1.06	1.42	0.42	0.33	1	0.0009
Coiled principal piece	7.17	7.56	6.92	9.42	7.39	8.61	8.28	10.22	8.2	0.0014
Rolled principal piece	0.86	0.36	0.92	0.53	0.92	0.75	1.25	0.86	0.81	0.0888
Bent principal piece	1.92	1.33	3.94	3.06	2.11	1.25	1.03	1	1.96	<0.0001
Broken principal piece	0.78	0.56	0.28	0.39	0.53	1.22	0.44	0.19	0.55	0.0002
Total principal piece	10.72	9.81	12.06	13.39	10.94	11.83	11.00	12.28	11.50	0.119
Plasma droplets at neck	1.47	6.72	1.11	0.81	0.97	3.22	1.31	7.5	2.89	<0.0001
Plasma droplets at midpiece	3.08	3.47	1.83	1.56	1.08	2.06	1.75	4.97	2.48	<0.0001
Plasma droplets at principal piece	0.06	0.14	0.03	0	0.03	0	0	0	0.03	0.002
Total plasma droplet	4.61 ^a^	10.33 ^b^	2.9 ^c^	2.36 ^c^	2.08 ^c^	5.28 ^a^	3.06 ^c^	12.47 ^b^	5.39	<0.0001
Individual heads	3.83 ^a^	2.5 ^b^	3.75 ^a^	3.19 ^ab^	1.56 ^c^	3.08 ^a^	1.58 ^c^	1.08 ^c^	2.57	<0.0001

Note: Data were organized and evaluated by two blind observers (400× objective). Values are reported as means (%). Statistical analysis used a Poisson GLMM (*glmmPQL* in R) with random effects; significance was assessed by Wald chi-square (*p* ≤ 0.05 significant; *p* ≤ 0.01 highly significant). Superscript letters indicate pairwise differences between staining methods (Tukey post hoc test). “Total” rows represent summed subcategories within each anatomical region. Methyl Violet was excluded due to staining instability. Abbreviations: E, Eosin; EN, Eosin–Nigrosin; DQ, Diff-Quick^®^; H, Hemacolor^®^; S, Spermac^®^; SG, Sangodiff-G^®^; FB, Formol–Citrate–Rose Bengal; TS, Testsimplets^®^; (*) Indicates statistical significance at *p* < 0.05.

**Table 3:** Description of sample staining results across different storage temperatures and analysis time points (1 = immediately, 2 = after one day, 3 = after one week, 4 = after three months) (*n* = 36).

Methods	Time 1	Time 2	Time 3	Time 4
Hemacolor	Pale color, fair detail visibility, good contrast across	Pale color, fair detail visibility, good contrast	Pale color, fair detail visibility, good contrast	16.67% partially assessable, 33.33% with colored particles, 16.67% with yellowish areas.
Sangodiff-G^®^	Pale color, fair detail, poor contrast; 50% granular background, 25% stained head caps, 16.67% colored particles	Pale color, fair detail, poor contrast; 50% granular background, 25% stained head caps, 16.67% colored particles	Analyzability dropped to 91.67%	Analyzability dropped to 55.56%
Formol– Citrate–Rose Bengal stain	100% Analyzable	100% Analyzable	100% Analyzable	94.44% Analyzable
Testsimplets^®^	100% Analyzable	97.22% Analyzable	72.22% Analyzable	11.11% Analyzable
Methyl Violet	100% Analyzable	100% Analyzable	0% Analyzable	0% Analyzable

Note: Data were organized and evaluated by two blind observers (400× objective). Values indicate qualitative staining quality or the percentage of analyzable slides at each time point. Statistical analysis used a Poisson GLMM (*glmmPQL in R*) with random effects; significance was tested using Wald chi-square (*p* ≤ 0.05 significant; *p* ≤ 0.01 highly significant).

**Table 4:** Mean percentages of individual pathomorphological characteristics across all staining methods, categorized by storage temperature at Evaluation Time 1. The data reflects trends associated with temperature effects and are not intended to evaluate the performance of specific staining methods.

Criteria	Temperature			
	18 °C	38 °C	6 °C	*p*-Value
Morphological abnormalities	30.59	31.34	31.26	0.908
Irregular head shape	0.48	0.33	0.56	0.047
Round head	0.38	0.41	0.32	0.626
Lanceolate head	9.91	8.9	8.95	0.526
Pear-shaped head	0.02	0.02	0.08	<0.0001
Double head	0.05	0.01	0.03	0.0238
Acrosome size variations (large/small)	0	0	0.01	0
Absence of acrosome	0.34	0.25	0.28	0.29
Acrosome detachment	2.06	2.21	1.73	0.18
Acrosome with vacuoles	0	0.06	0.06	0.814
Total head/acrosome	13.23	12.19	12.03	0.548
Asymmetrical midpiece	0	0	0	*
Broken midpiece	1.05	0.76	0.93	0.467
Thickened/narrow midpiece	0.06	0.02	0.06	0.0519
Total midpiece	1.1	0.78	0.98	0.398
Coiled principal piece	6.78	7.56	8.11	0.0629
Rolled principal piece	0.48	0.83	0.83	0.0191
Bent principal piece	1.83	1.83	1.8	0.986
Broken principal piece	0.42	0.64	0.41	0.0595
Total principal piece	9.51	10.86	11.15	0.0721
Plasma droplets at neck	2.56	2.67	2.47	0.842
Plasma droplets at midpiece	2.25	2.33	2.04	0.637
Plasma droplets at principal piece	0.05	0.01	0.03	0.0007
Total plasma droplet	4.86	5.01	4.54	0.662
Individual heads	1.89	2.5	2.56	0.0975

Note: Data were organized and evaluated by two blind observers (40× objective). Values represent mean percentages across all staining methods at Evaluation Time 1. Statistical analysis used a Poisson GLMM (*glmmPQL in R*) with random effects; significance was assessed by Wald chi-square (*p* ≤ 0.05 significant; *p* ≤ 0.01 highly significant). Parameters such as double heads and cytoplasmic droplets were considered morphologically constant and independent of storage conditions. Reported *p*-values indicate differences across temperatures only and do not imply dynamic changes in inherently fixed defects; ^®^; (*) Indicates statistical significance at *p* < 0.05.

**Table 5:** Mean slide processing times (minutes ± SD) and comparison of eosin and eosin–nigrosin staining results, including vitality assessment under different storage conditions. Values are presented as mean ± SD.

Staining Method	Preparation	Evaluation	Total Time	Vitality (%)	Vitality (Mean ± SD)	Total Exclu. Vitality (%)	Total Incl. Vitality (%)
Eosin	3.17 ± 10.42	5.00 ± 12.78	8.17 ± 0.23.2 *	RT: 80.01 ± 12.23			
				WB: 70.68 ± 16.10			
				RF: 84.97 ± 8.96	RT: 84 ± 5	RT: 82.12	RT: 84.67
					WB: 72 ± 6	WB: 71.02	WB: 72.14
					RF: 88 ± 4	RF: 85.34	RF: 88.11
Eosin–Nigrosin	3.67 ± 10.42	6.70 ± 11.65	10.37 ± 22.07 *	RT: 87.28± 7.87			
				WB: 79.27 ± 9.72			
				RF: 84.36 ± 8.49			
Diff-Quick^®^	5.73 ± 11.97	5.03 ± 8.67	10.76 ± 20.94				
Hemacolor^®^	5.73 ± 15.28	5.03 ± 8.67	10.76 ± 23.95				
Spermac^®^	12.64 ± 10.09	6.90 ± 12.16	19.54 ± 22.25				
Sangodiff-G^®^	14.43 ± 8.02	5.20 ± 9.96	19.63 ± 17.98				
Formol–Citrate–Rose Bengal	45.49 ± 1.72	6.48 ± 10.96	51.97 ± 12.32				
Methyl Violet	60.46 ± 0.89	4.98 ± 5.82	65.44 ± 8.55				
Testsimplets^®^	60.22 ± 2.73	7.45 ± 18.95	67.67 ± 19.84				

Note: Data was organized and evaluated by two blind observers (40× objective). Values represent mean durations (minutes ± seconds) for preparation, evaluation, and total processing of a stained slide. For total time, the additional assessment durations (4.15 ± 11.65 min for eosin and 4.97 ± 7.99 min for eosin–nigrosin) must be added to the corresponding totals, as these assessments are specific to these methods. Adjusted totals are reported accordingly. Time measurements were analyzed descriptively without statistical testing; RT = room temperature; WB = water bath; RF = refrigerator; SD = standard deviation; Total excl. vitality = total sperm parameters excluding vitality; Total incl. vitality = total sperm parameters including vitality assessment; ^®^; (*) Indicates statistical significance at *p* < 0.05.

**Table 6:** Cost analysis of staining methods/slides ranked by ascending total costs in Euros (€).

Staining Method	Material Components	Materials (€)	Total Time (min)	Labor (€)	Total (€)	Cost Variation (€)
Formol–Citrate–Rose Bengal	0.3 mL stain solution, 1 slide, 1 coverslip, 2 pipette tips	0.15	51.97	21.65	21.80	19.47–24.95
Methyl Violet	0.05 mL Methyl Violet, 1 slide, 1 pipette tip	0.17	65.44	27.27	27.44	24.54–30.71
Eosin	mL Eosin solution, 1 slide, 2 pipette tips	0.19	12.32	5.13	5.32	4.79–5.85
Eosin–Nigrosin	0.1 mL Eosin–Nigrosin solution, 2 slides, 2 pipette tips, 0.05 mL immersion oil	0.20	15.35	6.39	6.59	5.93–7.25
Diff-Quick^®^	3 × 1.66 mL staining solution, 2 slides, 1 pipette tip	1.22	10.76	4.49	5.71	5.14–6.28
Spermac^®^	4 × 0.25 mL staining solution, 2 slides, 1 pipette tip	0.90	19.54	8.15	9.05	8.14–10.01
Hemacolor^®^	3 × 0.5–2 mL staining solution, 2 slides, 1 pipette tip	0.46–1.36	10.76	4.49	4.95–5.85	4.46–6.43
Sangodiff-G^®^	stain with fixative solution, 2 slides, 1 pipette tip	0.83	19.63	8.18	9.01	8.11–9.91
Testsimplets^®^	1 pre-stained slide/coverslip, 1 pipette tip, 0.05 mL immersion oil	1.70	67.67	28.19	29.89	27.08–32.78

Note: Data were organized and evaluated by two blinds observers (40× objective). The table presents a cost analysis per stained slide, including reagents, slides, coverslips, and pipette tips. Total time is the sum of preparation and evaluation per slide. Labor costs were calculated from total processing time using an hourly wage of €25 for a certified veterinary technician. Total (€) reflects materials plus labor; cost variation (€) accounts for ±10% changes in material prices and the inclusion/exclusion of labor costs.

## Data Availability

The authors retain ownership of the data produced in this study, which can be obtained by contacting the corresponding author.
